# Validated DBS method for filgotinib quantitation in rat dried blood spots and its application to a pharmacokinetic study in rats

**DOI:** 10.5599/admet.796

**Published:** 2020-05-06

**Authors:** Abhishek Dixit, Vinay Kiran, Bhavesh Babulal Gabani, Ramesh Mullangi

**Affiliations:** Drug Metabolism and Pharmacokinetics, Jubilant Biosys Ltd, Industrial Suburb, Yeshwanthpur, Bangalore-560 022, India

**Keywords:** Filgotinib, LC-MS/MS, method validation, rat blood, DBS, pharmacokinetics

## Abstract

Filgotinib is a selective JAK1 (Janus kinase) inhibitor, showed efficacy in patients suffering from moderate-to-severe rheumatoid arthritis. In this paper, we present the data on the development and validation of a sensitive, selective and high-throughput LC-MS/MS (liquid chromatography with tandem mass spectrometry) method for the quantitation of filgotinib from rat dried blood spot (DBS) cards. To the DBS disc cards, 0.2% formic acid enriched with internal standard (IS) was added and sonicated. Thereafter the extraction of filgotinib and the IS (tofacitinib) was accomplished using ethyl acetate as an extraction solvent. The resolution of filgotinib and the IS was achieved on a Gemini C_18_ column with an isocratic mobile phase, which is a mixture of 0.2% formic acid:acetonitrile (20:80, v/v) at a flow-rate of 0.9 mL/min. The total run time was 2.90 min and the retention time of filgotinib and the IS was ~1.31 and 0.89 min, respectively. Filgotinib and the IS were analyzed using positive ion scan mode and parent-daughter mass to charge ion (m/z) transition of 426.3→291.3 and m/z 313.2→149.2, respectively, for quantitation. The calibration range was 1.37-1937 ng/mL. No matrix effect and carry over were observed. All the validation parameters met the acceptance criteria. The validated method has been applied to a pharmacokinetic study in rats. A good correlation between DBS and plasma concentrations for filgotinib was observed.

## Introduction

**Figure 1.**
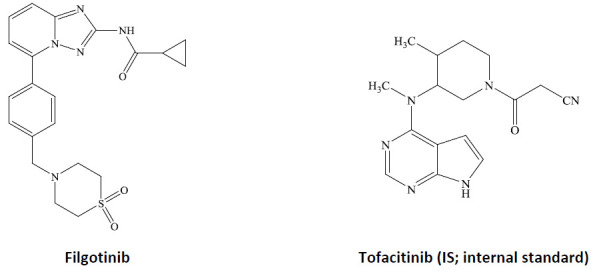
Structural representation of filgotinib and tofacitinib (IS).

Rheumatoid arthritis is a chronic autoimmune inflammatory disease with a prevalence of 1-2% of the world population. Rheumatoid arthritis primarily affects peripheral joints and subsequently damages the synovial tissue and cartilage damage [1]. Although disease-modifying anti-rheumatic drugs (DMARDs: methotrexate, sulfasalazine, leflunomide etc) and biologic DMARDs (etanercept, adalimumab etc.) are available as first-line drugs due to their low therapeutic benefit and severe side effects they cannot be used for long-treatment [2]. To overcome these drawbacks, Janus kinase (JAK)/signal transducer and activator of the transcription (STAT) signal pathway has been identified as one of the new therapeutic targets to treat rheumatoid arthritis. JAK-STAT pathway plays a critical role in the downstream signaling of cytokines. The inhibition of JAKs is an attractive therapeutic target to treat rheumatoid arthritis [3]. Tofacitinib and baricitinib are the pan-JAK inhibitors (JAK1/JAK3) approved for the treatment of moderate to severe rheumatoid arthritis but they showed dose-related toxicities [4]. Recent findings suggest that selective inhibition of JAK1 might reduce the toxicity without a significant detriment to efficacy [2, 3]. Filgotinib (Figure 1; GLPG0634), is a selective JAK1 inhibitor (IC_50_: 629 nM) with 30-fold selectivity over JAK2 [5]. It has shown dose-dependent reduction of disease progression in a collagen-induced arthritis model post oral administration in rodents [5]. In Phase-3 clinical trials, filgotinib was well tolerated, showed efficacy and was found to be safe in rheumatoid arthritis patients, when it was administered as monotherapy at 100 or 200 mg, once daily or with methotrexate [6]. Filgotinib is currently being filed in the US and Japan to treat the patients suffering from moderate-to-severe rheumatoid arthritis [7, 8].

To date, two LC-MS/MS (liquid chromatography with tandem mass spectrometry) methods were reported for the quantification of filgotinib. The first method, reported by Namuor et al. (2015) employed solid-phase extraction method for the quantitation of filgotinib and its active metabolite from Phase-I study plasma samples [100 μL plasma sample was spiked with 20 μL of deuterated filgotinib (125 ng/mL) as an internal standard]. The reported lower limit of quantification (LLOQ) was 3.00 ng/mL. However, full details on chromatography, mass spectrometer conditions and validation parameters were not reported [9]. Very recently, Dixit et al. (2020) reported a validated LC-MS/MS method for the quantification of filgotinib. The authors have attained an LLOQ of 0.78 ng/mL with 50 μL rat plasma. Plasma samples were processed using ethyl acetate as an extraction solvent [10].

Dried blood spot (DBS) methodology has seen significant progress during recent times in the quantitative analysis of various drugs [11-16]. DBS offers several advantages over traditional sampling (plasma/blood/serum) techniques such as reduction of commercial costs for laboratory equipment, convenience in collection, reduction in collection of blood volume, no requirements for trained phlebotomist, ease of sample handling/storage/shipping, safety in handling, less time in processing and increase in throughput etc. It is anticipated that much clinical development and therapeutic drug monitoring programs in the future may switch to DBS technique to characterize the pharmacokinetic data.

To the best of our knowledge, there is no DBS method reported for the quantitation of filgotinib. In this paper, we report the development and validation of an LC-MS/MS method for the quantitation of filgotinib on rat DBS. The applicability of the validated method was shown in a rat pharmacokinetic study. Excellent correlation was observed between DBS *versus* plasma filgotinib concentrations, indicating that the DBS method can be used as an alternative for plasma sampling for pharmacokinetic analysis.

## Materials and methods

### Materials

Filgotinib (purity: >95%) was purchased from Angene International Limited, Tsuen Wan, Hong Kong. Tofacitinib (IS; purity: 98%) was purchased from Sigma-Aldrich (St. Louis, USA). HPLC grade acetonitrile and methanol were purchased from J.T. Baker, PA, USA. Analytical grade formic acid was purchased from S.D Fine Chemicals, Mumbai, India. All other chemicals and reagents were of analytical grade and used without further purification. The control Sprague Dawley rat blood was procured from Animal House, Jubilant Biosys, Bangalore.

### Liquid chromatography and mass spectrometry conditions

We used the similar chromatographic conditions that we previously reported [10]. In brief, a Gemini C18 (100 × 4.6 mm, 3 μm) column maintained at ambient room temperature along with an isocratic mobile phase (0.2% formic acid in Milli-Q water:acetonitrile, 20:80, v/v) at a flow-rate of 0.9 mL/min was used for separation of filgotinib and the IS. The injection volume was 5.0 μL. Under these optimized conditions the retention times were 1.31 and 0.89 min, for filgotinib and the IS, respectively with a total run time of 2.90 min. The LC was coupled to a Sciex 4000 mass spectrometer controlled by Sciex Analyst 1.6.2 software. The MS was equipped with electro-spray ionization and operated in the multiple reaction monitoring (MRM) mode for the quantitation. Ionization was conducted by applying a voltage of 5500 V, and the source temperature was set at 550 °C. The gas settings were as follows - curtain gas: 35 psi, GS1: 50 psi, GS2: 55 psi and CAD: 8.0 psi. The compound parameters declustering potential, entrance potential, collision energy, and collision cell exit potential were set at 91, 10, 40, and 10 V for filgotinib and 100, 10, 41, and 12 V for the IS. The mass transitions *m/z* (precursor ion→product ion) 426.3→291.3 and 313.2→149.2 were monitored for filgotinib and the IS, respectively. Quadrupole Q1 and Q3 were set on the unit resolution. Dwell time was 150 ms.

### Preparation of stock solutions and standard samples

Two separate primary stock solutions of filgotinib were prepared at 1670 μg/mL in DMSO. Appropriate secondary and working stocks of filgotinib were prepared from primary stock by successive dilution of primary stock with methanol:water (80:20, v/v) to prepare the calibration curve (CC) and quality controls (QCs). The IS primary stock solution was made in DMSO at a concentration of 1000 μg/mL, which was diluted with methanol to 500 ng/mL and subsequently used as IS working stock solution. The primary stock solutions of filgotinib and the IS were stored at -20 °C, which were found to be stable for 50 days. Working stock solutions were stored at 4 °C for 25 days.

### Blood spotting

Freshly drawn rat blood was used to prepare the DBS cards. With the help of a calibrated pipette, 25 μL of the respective spiked CC/QC blood or whole blood collected from the pharmacokinetic study (post administration of filgotinib by *intravenous* and oral routes) was sampled on DBS card. Spiked cards were allowed to dry at ambient room temperature for 3 h and stored appropriately in a sealed bag with desiccant in a desiccator.

### DBS homogeneity

The spot homogeneity was evaluated by punching out the disc from the periphery of the DBS. Blood spots at QC low and QC high level were prepared in triplicate. The obtained DBS discs were processed and analyzed as described in the Sample Preparation section.

### DBS sample preparations

Using a hole puncher (Harri-Micro-Punch®), 3 mm diameter disc was punched from the center of each DBS (FTA DMPK-C Card) card directly into micro-centrifuge tubes. To each micro-centrifuge tube, 200 μL of 0.2% formic acid enriched with 100 ng/mL of IS was added, thereafter the contents were vortex mixed for 5 min (Thermomixer^®^, Eppendorf) and sonicated (Elmasonic S300 H) for 15 min at ambient room temperature. After sonication, to the same micro-centrifuge, 1.0 mL of ethyl acetate was added and the mixtures were vortexed further for 3 min. The samples were centrifuged at 14,000 rpm for 3 min. Clear organic layer (800 μL) was pipetted out after centrifugation and dried under gentle stream of nitrogen (Turbovap^®^, Zymark^®^,Kopkinton, MA, USA) at 50 °C. The residue was reconstituted with 200 μL of 0.2% formic acid in acetonitrile and 150 μL clear supernatant was aliquoted into a HPLC vial and 5.0 μL was injected onto the column for LC-MS/MS analysis.

### Validation procedure

The validation experiments were performed in accordance with the US Food and Drug Administration guideline [17]. Various parameters covered under validation are: selectivity, carry over (repetitive punching and auto-injector), recovery, matrix effect, linearity, precision, accuracy, stability (in-injector, 6 h at room temperature and 30-day long-term at -80 ± 10 °C) and incurred sample reanalysis.

### Influence of hematocrit

Two different hematocrit (Hct) blood samples at 30 and 50% were prepared at LQC and HQC by adding or removing the plasma to the rat blood. These samples (in quadruplicate at each QC) were analysed with calibrators prepared in blood at standard fixed 40% Hct. The relative error of ±15% and precision of ±15% was considered acceptable. Hct value in rats ranged between 38-46% (in-house data; measured using Mindray BC-5000Vet) and it is in line with reported values [18].

### Pharmacokinetic study in rats

All the animal experiments were approved by Institutional Animal Ethical Committee (IAEC/JDC/2019/189R). Male Sprague Dawley rats (n=8) were procured from Vivo Biotech, Hyderabad, India. The animals were housed in Jubilant Biosys animal house facility in a temperature (22 ± 2 °C) and humidity (30-70%) controlled room (15 air changes/hour) with a 12:12 h light:dark cycles, had free access to rodent feed (Altromin Spezialfutter GmbH & Co. KG., Im Seelenkamp 20, D-32791, Lage, Germany) and water for one week before using for experimental purpose. Following ~12 h fast (during the fasting period animals had free access to water) animals were divided into two groups having four rats in each group. Group I animals (205-210) received filgotinib orally as a suspension formulation (prepared using Tween-80 and 0.5% methyl cellulose) at 10 mg/kg (strength: 1.0 mg/mL; dose volume: 10 mL/kg), whereas Group II animals (215-225 g) received filgotinib *intravenously* (5% DMSO, 5% Solutol:absolute alcohol (1:1, v/v) and 90% of normal saline; strength: 0.5 mg/ml; dose volume: 4 ml/kg) at 2.0 mg/kg dose. Post-dosing serial blood samples (50 μL) were collected through retro-orbital plexus into polypropylene tubes containing K_2_.EDTA solution as an anti-coagulant at 0.25, 0.5, 1, 2, 4, 8, 10, 12 and 24 h (for oral study) and 0.083, 0.25, 0.5, 1, 2, 4, 8 and 24 h (for *intravenous* study). Animals were allowed to access feed 2 h post-dosing.

### Pharmacokinetic analysis

Blood concentration-time data of filgotinib was analyzed by non-compartmental method and the relevant pharmacokinetic parameters namely AUC_0-∞_ (area under the blood concentration-time curve from time zero to infinite time), *C*_0_ (extrapolated blood concentration at time zero), *C*_max_ (maximum blood concentration), *T*_max_ (time to reach *C*_max_), *V*_d_ (volume of distribution), CL (clearance) and *t*_½_ (terminal half-life) were calculated using Phoenix WinNonlin software (version 8.1; Pharsight Corporation, Mountain View, CA). Absolute oral bioavailability (*F*) was calculated using this formula [Dose (*i.v.*) × AUC_(0-∞)oral_ / Dose (oral) × AUC_(0-∞)_*_i.v._*] × 100.

## Results and discussion

### Method development

The objective of this work was to develop a sensitive and rugged DBS method for the quantification of filgotinib in rat blood and subsequently show the correlation with previously developed method [10] in a pharmacokinetics study so that DBS method can be used as an alternative method to measure the filgotinib concentrations. Previously reported LC-MS/MS conditions were utilized for this method [10].

Since chromatography and mass spectrometric conditions were optimized, to obtain better sensitivity extraction optimization was done with various extraction solvents namely acetonitrile, methanol, ethyl acetate, acidified methanol/acetonitrile, mixture of water and methanol/acetonitrile, pre-treatment of DBS discs with formic acid followed by extraction with methanol/acetonitrile/ethyl acetate.

### Method validation parameters

#### Recovery

None of the single organic solvents and pure water gave good recovery of filgotinib from the DBS discs. The recovery with ethyl acetate, water and methanol/acetonitrile was 5, 39 and 43%, respectively. With methanol/acetonitrile:water 50:50 and 20:80, the recovery was found to be 30 and 43%, respectively. In order to improve the recovery, the DBS discs were pre-treated with 0.2% formic acid and extracted with ethyl acetate. This process helped to attain the recovery up to 62%. The mean ± S.D recovery of filgotinib at LQC and HQC was found to be 62.4 ± 8.39 and 64.2 ± 4.26%, respectively. The recovery of the IS was 95.2 ± 4.85%.

#### Matrix effect

The mean absolute matrix effect for filgotinib on rat DBS cards at LQC and HQC was found to be 0.81 ± 0.08 and 0.83 ± 0.08%, respectively. The matrix effect for the IS was 0.97 ± 0.05% (at 100 ng/mL). These results indicate that the minimal matrix effect did not obscure the quantification of on filgotinib and the IS from rat DBS cards.

#### Selectivity and carry over

**Figure 2.**
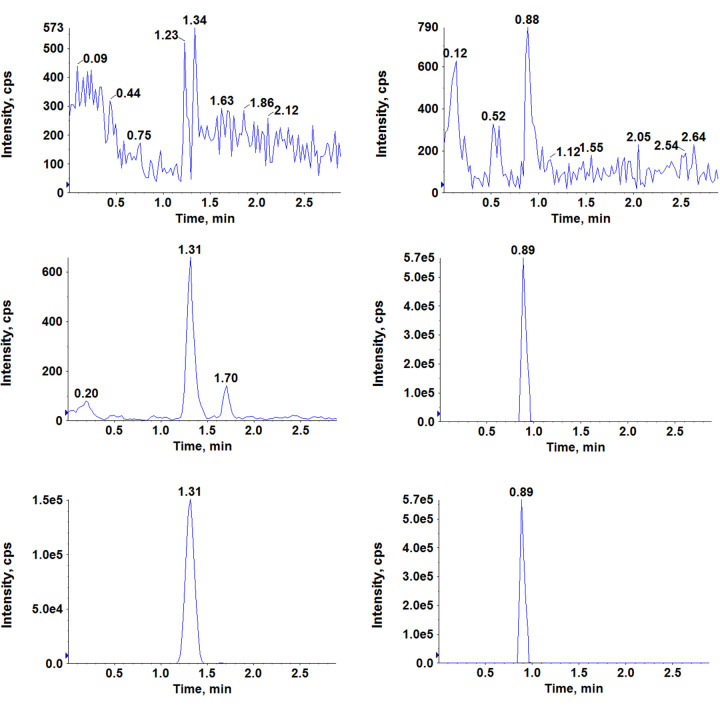
Typical MRM chromatograms of filgotinib (left panel) and the IS (right panel) in (a) rat blank DBS card (b) rat blank DBS card spiked with filgotinib at LLOQ (1.37 ng/mL) and the IS (c) a 0.25 h *in vivo* plasma sample showing filgotinib peak obtained following oral administration to rat along with the IS.

Figures 2a,b,c show chromatograms for the blank rat DBS cards (free of analyte and the IS; Figure 2a), blank rat DBS cards spiked with filgotinib at LLOQ and the IS (Figure 2b) and an *in vivo* blood sample obtained at 0.25 h after oral administration of filgotinib along with the IS (Figure 2c). There was no carry-over produced by the highest calibration sample on the following injected blank DBS extract sample. Additionally, no DBS-specific device-oriented carry-over was noted.

#### Calibration curve

The calibration curve was constructed in the linear range using eight calibrators 1.34, 2.67, 14.7, 26.7, 400, 801, 1603 and 1937 ng/mL. The typical regression equation for calibration curve was *y* = 0.000849 *x* + 0.00127. The correlation coefficient (r) average regression (*n*=4) was found to be >0.9966 for filgotinib. The lowest concentration with the RSD <20% was taken as LLOQ and was found to be 1.37 ng/mL. The accuracy observed for the mean of back-calculated concentrations for four calibration curves for filgotinib was within 95.9-110%; while the precision (CV) values ranged from 3.87-7.94%.

#### Accuracy and precision

Accuracy and precision data for intra- and inter-day DBS samples for filgotinib is presented in Table 1. The assay values on both the occasions (intra- and inter-day) were found to be within the accepted variable limits.

#### Stability

The predicted concentrations for filgotinib at 4.01 and 1736 ng/mL samples deviated within ±15% of the fresh sample concentrations in a battery of stability tests: bench-top (6 h), in-injector (21 h) and freezer stability at -80 ± 10 °C for at least for 30 days (Table 2). The results were found to be within the assay variability limits during the entire process.

#### Incurred samples reanalysis (ISR)

**Figure 3.**
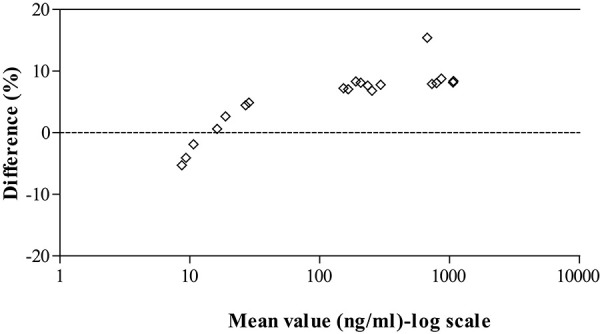
Bland-Altman plot showing the incurred sample re-analysis data for filgotinib on DBS.

As per guidance [17] around 10% of the study samples should be selected for ISR if the total sample size is less than 1000. In this validation a total of 20 samples were chosen for ISR from the oral and *intravenous* rat pharmacokinetic studies. From oral arm samples near *C*_max_ (0.5 h) and elimination phase (4 h and 24 h) were selected. However, for *intravenous* arm representative samples at 0.083, 2 and 8 h time points were selected. Figure 3 shows the comparison of ISR values *versus* original values using Bland-Altman plot suggesting that all the ISR samples were within ±20% of the original values.

#### Hematocrit effect

Hematocrit (Hct) has a significant effect on the viscosity of the blood, which influences the flux and diffusion properties of the blood through DBS card used for sample collection. It can directly affect the accuracy of the analysis in DBS samples. The reported blood-to-plasma ratio for filgotinib was 1.22 [19] indicating an almost equal plasma and red blood cell distribution. The measured filgotinib concentrations were compared with the results obtained from DBS samples are given in Table 3, indicating that Hct had no significant influence on the filgotinib concentration.

#### Pharmacokinetic study

**Figure 4.**
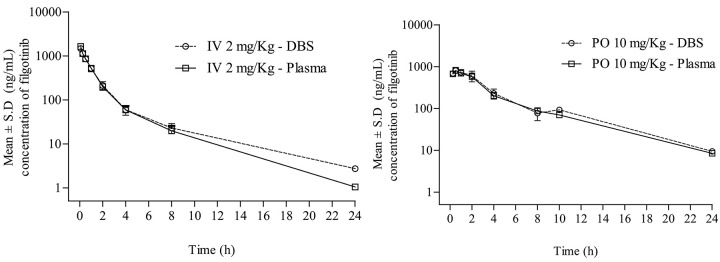
**a**) mean plasma concentration-time profiles of filgotinib in rat blood and plasma following *intravenous* administration of filgotinib; **b**) mean plasma concentration-time profiles of filgotinib in rat blood and plasma following oral administration of filgotinib.

The sensitivity of the present DBS method was found to be sufficient for accurately characterizing the pharmacokinetics of filgotinib by oral and *intravenous* routes in rats. To assure acceptance of study sample analytical runs, at least two-thirds of the QC samples had to be within ±15% accuracy, with at least half of the QC samples at each concentration meeting these criteria. Results indicated that QCs met the acceptance criteria. Figures 4a and 4b show the mean blood concentration *versus* time for filgotinib post administration of *intravenous* and oral route, respectively in rats. The pharmacokinetic parameters are summarized in Table 4 along with previously reported pharmacokinetic parameters by Dixit et al. [10]. Filgotinib was quantifiable up to 24 h post *intravenous* and oral administration to rats. Following *intravenous* administration at 2.0 mg/kg, the plasma concentrations decreased mono-exponentially. Filgotinib exhibited moderate clearance (Cl) of 18.7 ± 1.86 mL/min/kg, which is ~3-fold lower than hepatic blood flow (55 mL/min/kg) and volume of distribution (*V*_d_) was 4.17 ± 0.33 L/kg in rats. The terminal half-life was found to be 3.52 ± 0.23 h. Following oral administration filgotinib maximum plasma concentrations (*C*_max_: 804 ± 78.4 ng/mL) attained at 0.50 ± 0.00 h (*T*_max_) in all rats suggesting that filgotinib has a rapid absorption from the gastrointestinal tract. The AUC_0-∞_ (area under the plasma concentration-time curve from time zero to infinity) was found to be 3722 ± 329 and 1801 ± 188 ng×h/mL, by oral and *intravenous* routes, respectively. The terminal half-life (*t*_½_) determined after oral administration was 4.87 ± 0.45 h. The absolute oral bioavailability for filgotinib in rats at 10 mg/kg was 42.5%.

In Figures 4a and 4b the blood *versus* time concentration profiles of filgotinib obtained from *intravenous* and oral routes in rats were presented along with plasma *versus* time concentration profiles reported earlier by us [10] showed excellent correlation (R^2^ > 0.99). This indicates that DBS can be used as an alternative strategy for determination of filgotinib circulatory concentration in a pharmacokinetic study. To the best of our knowledge, to date there is no comparison between plasma and whole blood for filgotinib. Our study demonstrated that DBS is a promising alternative to plasma sampling for filgotinib in rheumatoid arthritis patients especially with elderly patients in remote or resource-limited settings or immobile patients.

## Conclusions

In summary, a simple and rapid method using LC-MS/MS for the determination of filgotinib in rat blood using DBS cards was developed and validated as per US FDA regulatory guideline. The validated method suitability was shown in a rat pharmacokinetic study.

## Figures and Tables

**Table 1.** Precision and accuracy determination of filgotinib quality controls in rat plasma

	LLOQ QC (1.37 ng/mL)	LQC (4.01 ng/mL)	MQC (1068 ng/mL)	HQC (1736 ng/mL)
*Intra-day (n=6)*				
Mean ± S.D	1.34 ± 0.10	4.30 ± 0.45	1136 ± 73.3	1715 ± 124
Precision (%RSD)	7.38	10.4	6.46	7.23
Accuracy (%RE)	1.00	1.07	1.06	0.99
*Inter-day (n=24)*				
Mean ± S.D	1.33 ± 0.15	3.94 ± 0.44	1064 ± 96.8	1663 ± 128
Precision (%RSD)	11.0	11.2	9.09	7.71
Accuracy (%RE)	1.00	0.98	1.00	0.96

RSD: relative standard deviation (SD ×100/Mean) RE: relative error (measured value/actual value)

**Table 2.** Stability data of filgotinib quality controls in rat plasma

Concentration spiked (ng/mL)	Bench-top for 6 h (n=6)		Long-term 30 days at -80 °C (n=6)		In-injector for 21 h (n=6)	
	% RE	%RSD	% RE	%RSD	% RE	%RSD
4.01	0.96	11.1	0.97	15.1	0.91	5.99
1736	0.88	4.09	0.96	6.04	0.88	2.39

RSD: relative standard deviation (SD ×100/Mean) RE: relative error (measured value/actual value)

**Table 3.** Haematocrit effect analysis

Concentration spiked (ng/mL)	Hematocrit (%)	Concentration found (ng/mL)	Accuracy (%RE)	Precision (% RSD)
4.01	30	3.95 ± 0.19	0.98	4.81
	50	3.90 ± 0.24	0.97	6.29
1736	30	1664 ± 80.4	0.95	4.83
	50	1786 ± 88.7	1.02	4.96

**Table 4.** Pharmacokinetic parameters for filgotinib in rats following oral and *intravenous* administration

PK parameters	Oral	*Intravenous*
Dose (mg/kg)	10	2.0
AUC_(0-∞)_ (ng × h/ml)	3722 ± 329 (3475 ± 547)	1801 ± 188 (1912 ± 123)
C_max_/C_0_ (ng/ml)	804 ± 78.4 (802 ± 149)	1602 ± 55.5 (1767 ± 128)
T_max_ (h)	0.50 ± 0.00 (0.50 ± 0.00)	---
T_1/2_ (h)	4.87 ± 0.45 (4.72 ± 0.24)	3.52 ± 0.23 (3.56 ± 0.30)
CL (ml/min/kg)	---	18.7 ± 1.86 (17.4 ± 1.08)
Vd (L/kg)	---	4.17 ± 0.33 (5.41 ± 0.77)
F (%)	42.5 (36.3)	---

The values in parentheses are reported by Dixit et al. (2020) [10].
